# Personalized tobramycin dosing in children with cystic fibrosis: an AUC_24_-guided approach

**DOI:** 10.1128/aac.00278-25

**Published:** 2025-07-23

**Authors:** Kiera H. Harwood, Stephen Duffull, Shivanthan Shanthikumar, Alice Lei, Sarath Ranganathan, Phil Robinson, Indy Sandaradura, Tony Lai, Amanda Gwee

**Affiliations:** 1 Department of Paediatrics, The University of Melbourne 569523, , Parkville, Victoria, Australia; 2 Antimicrobials Group, Murdoch Children's Research Institute 34361, Parkville, Victoria, Australia; 3 Certara 10771 , Princeton, New Jersey, USA; 4 Department of Respiratory and Sleep Medicine, Royal Children's Hospital 6453, Parkville, Victoria, Australia; 5 Respiratory Diseases Group, Murdoch Children's Research Institute 34361, Parkville, Victoria, Australia; 6 The Children's Hospital at Westmead 8538, Sydney, New South Wales, Australia; 7 University of Sydney 4334, Sydney, New South Wales, Australia; 8 Department of General Medicine, Royal Children’s Hospital 545982 , Parkville, Victoria, Australia; University of Pittsburgh School of Medicine, Pittsburgh, Pennsylvania, USA

**Keywords:** population pharmacokinetics, cystic fibrosis, aminoglycosides

## Abstract

Tobramycin is commonly used for the treatment of pulmonary exacerbations in children with cystic fibrosis (CF). Currently, a standard dose of 10 mg/kg daily is used in all children. We aim to develop a population pharmacokinetic (popPK) model of tobramycin in children with CF and determine the: (i) effect of cystic fibrosis transmembrane conductance regulator (CFTR) modulators on tobramycin pharmacokinetics (PK); (ii) attainment of the commonly used serum steady state area under the concentration-time curve target (AUC_24,ss_) of 80–110 mg/L⋅h with standard dosing; and (iii) generate an optimized fully individualized dosing strategy to improve target attainment. Multicenter prospective observational study of children with CF aged 0–19 years receiving IV tobramycin who had ≥1 serum concentration measured. A popPK model was developed using nonlinear mixed-effect modeling, and simulations were performed to assess study aims. Overall, 63 children had 450 serum tobramycin concentrations. A one-compartment popPK model, including age, weight, a renal maturation model, and estimated glomerular filtration rate as covariates, was developed. With standard dosing, 1/3 of children achieved the target AUC_24,ss_ with younger children (<2 years) having the lowest probability of target attainment (PTA) (15%). The optimized dosing regimen improved target attainment in all children, increasing the PTA in children <2 years to 62%. CFTR modulator drugs did not affect tobramycin PK. Standard tobramycin dosing in children with CF achieves poor attainment of target serum AUC_24,ss_, particularly in children <2 years. A fully individualized approach (available at https://www.kidscalc.org/) improved target attainment in all children. CFTR modulators had a negligible effect on tobramycin PK.

## INTRODUCTION

Tobramycin is one of the most commonly used antibiotics used to treat pulmonary exacerbations in children with cystic fibrosis (CF) infected with *Pseudomonas aeruginosa* (1). It exhibits concentration-dependent bactericidal activity (i.e., a higher peak concentration is associated with increased bacterial killing) against aerobic Gram-negative bacteria; however, excess drug accumulation results in increased adverse effects (nephrotoxicity, ototoxicity). As a result, trough concentrations, with or without peak concentrations, have traditionally been measured for therapeutic drug monitoring (TDM) (2, 3). More recently, animal and clinical studies have shown that the ratio of the steady state serum area under the concentration-time curve over 24 h to the minimum inhibitory concentration of the bacteria (AUC_24,ss_/MIC) correlates with bactericidal activity, clinical improvement, and risk of toxicity (3–6). Therefore, many CF centers have moved to AUC_24,ss_-guided dosing for tobramycin (7–9).

A commonly used therapeutic target for tobramycin is a serum AUC_24,ss_/MIC ratio of ≥80 with studies reporting a greater improvement in pulmonary function in those who achieved this compared with those who did not (6, 10, 11). However, as MIC testing is often not routinely performed for *P. aeruginosa* isolates in patients with CF, an AUC_24,ss_ target of 80–120 mg/L⋅h is often used as a surrogate (3, 4, 12, 13). An upper limit of 120 mg/L⋅h is commonly implemented in clinical practice based on several clinical studies showing increased risk of nephrotoxicity/ototoxicity associated with increasing tobramycin exposure (2, 7, 14, 15). Previous pediatric pharmacokinetic (PK) studies showed that with standard dosing of 10 mg/kg once daily (OD), less than half of children achieved a target AUC_24,ss_ of 80–115 mg/L⋅h (4, 13). This highlights the need to improve tobramycin dosing, particularly in those with chronic *P. aeruginosa* infection.

The licensing of cystic fibrosis transmembrane conductance regulator (CFTR) modulator drugs has led to reduced pulmonary exacerbations, morbidity, and increased survival in those with CF (16, 17). There are several CFTR modulators (ivacaftor, lumacaftor/ivacaftor, tezacaftor/ivacaftor, and elexacaftor/tezacaftor/ivacaftor) now licensed for use in children with CF. As the CFTR channel is highly expressed in the kidney, it is commonly thought that channel dysfunction seen in CF patients may affect renal function, leading to enhanced glomerular filtration in these individuals (18–23). Therefore, it is expected that those receiving CFTR modulator treatment will display altered renal function affecting tobramycin PK compared with those who are not.

This study aimed to develop a population pharmacokinetic model (popPK) of tobramycin in children with CF and determine (i) the effect of concomitant CFTR modulators on tobramycin PK; (ii) the attainment of an AUC_24,ss_ of 80–110 mg/L⋅h with standard dosing; and (iii) the attainment of this therapeutic target using a fully individualised dosing strategy in children with CF.

## MATERIALS AND METHODS

### Patients and study design

This multicenter prospective observational study collected data from children with CF receiving IV tobramycin at The Royal Children’s Hospital (RCH), Melbourne, and The Children’s Hospital at Westmead (CHW), Sydney, over a 2-year period (August 2020 and August 2022). All children aged 0 to 19 years of age with CF who received IV tobramycin and had at least one tobramycin concentration measured were included. Ethics approval was obtained from RCH Human Research Ethics Committee (HREC65652), and due to the observational study design, consent was not required from patients/families. Tobramycin dosing and TDM were performed in accordance with institutional guidelines. Children admitted to RCH received an initial dose of 10 mg/kg OD and had serum concentrations determined 1–2 h and 4–8 h after the start of the infusion to determine the AUC_24,ss_ (24). Children admitted to CHW received an initial dose of 10–12 mg/kg OD with a serum concentration determined 2–6 h after a dose (9). Additional TDM was conducted as per routine care, often after a dose change, change in serum creatinine, or weight, which may influence tobramycin PK. Doses were infused over 30 min at both centers.

Eligible patients were identified through a daily report in the hospitals’ electronic medical record system (EPIC), and data from patient files were both electronically and manually extracted. These data included patient demographics (age, height, weight), clinical details (CFTR mutation, serum creatinine and albumin levels, change in lung function), tobramycin dosing and all available drug monitoring data, and concomitant medications (antimicrobials, CFTR modulator drugs, diuretics, non-steroidal anti-inflammatory drugs [NSAIDs]).

Tobramycin concentrations were determined using an accredited competitive immunoassay (VITROS XT 7600, Chemistry Products TOBRA Reagent, QuidelOrtho, Corp., San Diego, CA at RCH, and VITROS 5600 at CHW), both of which were subject to external quality assurance. The lower limit of quantification was 0.6 mg/L. Concentrations below the limit of quantification (BLQ) were handled using the M6 method where the first in a sequence of BLQ values were replaced with 0.3 mg/L (half the quantification limit) (25). If multiple BLQ measurements occurred after a single dose, only the first was retained, and subsequent values were excluded from the analysis.

### Population pharmacokinetic analysis

Data were analyzed using a nonlinear mixed effect modeling approach in NONMEM version 7.4.3 (NONMEM, ICON Development Solutions, Ellicott City, MD) with an Intel FORTRAN compiler and PsN (Perl-speaks-NONMEM) version 5.3.0. The first-order conditional estimation method with interaction was used for all analyses.

Model development was based on a three-stage process: (i) determination of the best structural model (compartmental, between-subject variability, and residual variability); (ii) inclusion of covariate models to account for maturation and size; and (iii) evaluation of further covariates. Statistical significance for nested models was based on a likelihood ratio test where a drop in the objective function value (OFV) of greater than 3.84 units corresponds to a *P*-value of <0.05. The OFV provided by NONMEM is proportional to −2log likelihood (−2LL), and the difference of two −2LL is approximately and asymptotically Chi-squared distributed. For non-nested models with the same number of estimated parameter values, the model with the lower OFV was accepted (this is equivalent to the Akaike Information Criterion).

Covariates evaluated included size as measured by weight (WT) or fat-free mass (estimated using methods described in reference 26), estimated glomerular filtration rate (eGFR using the modified Schwartz equation [27]), serum creatinine, serum albumin, concomitant medications (CFTR modulators, NSAIDs, diuretics), and renal maturation. The latter was derived from a previously published model that predicts clearance based on body size and post-menstrual age (PMA), with the Hill coefficient and TM50 parameter values for GFR extracted from Table 3 of the paper (28). Continuous covariates were scaled to the median value for the population and added in a stepwise fashion to the model. Furthermore, WT and fat-free mass were evaluated by either fixing the exponent to 0.75 and 1 for clearance (CL) and volume of distribution (V), respectively, or by estimating the exponent. Further information regarding the handling of covariates is described in the Supplemental material.

Inclusion of covariates was assessed using forward stepwise evaluation based on statistical significance (OFV drop of 3.84 or greater), model stability (e.g., successful covariance step from NONMEM), biological plausibility, and clinical importance. Xpose in R was used for model comparison and to assess the accuracy of the final model using goodness of fit plots and visual predictive checks.

Simulations were performed using R Studio version 4.3.0. The input-output model used for the simulations was based on the final model. Covariates from a separate database of 704 children aged 0.1 to 19.3 years, with 1,489 admissions, were used to generate the virtual patients, which displayed a full range of covariates in this study where all patients had age-related normal serum creatinine concentrations. Virtual patients were re-sampled with replacement from this database, and the final popPK model was used to calculate the AUC_24,ss_ with standard dosing of 10 mg/kg/day and individualized dosing. Standardized virtual patients were constructed to calculate the CL based on the PK covariate model, which calculated the dose required to achieve a target AUC_24,ss_. The virtual patients were then individualized by adding random error (based on the interindividual variability omega) to calculate their individual CL, from which the actual AUC_24,ss_ was calculated using the equation: ${AUC}_{24,ss,}=Dose/CL$. This provided the theoretical best probability of target attainment (PTA). The target AUC_24,ss_ used for the theoretical best PTA was 93.8 mg/L⋅h. The individualized CL was also used to calculate the AUC_24,ss_ from standard dosing, where the dose was fixed at 10 mg/kg (1, 2, 29). For the individualized dosing regimen, the optimal dose was calculated using standardised CL and a target AUC_24,ss_ of 95 mg/L⋅h. Dosing was capped according to RCH guidelines of a maximum daily dose of 850 mg or 16 mg/kg, whichever was the lowest. The actual AUC_24,ss_ was then calculated based on the optimal dose and individualized CL. The probability of target attainment (PTA) of achieving a target AUC_24,ss_ between 80 and 110 mg/L⋅h with the standard as well as the individualized regimen was calculated and compared with the theoretical best PTA. Simulated patients were also stratified into age groups (<2 years, 2 to 12 years, and >12 years). To generate robust statistical analyses, 1,000 virtual patients for each age group were generated, and the PTA from standard and optimized dosing within each age group was calculated and compared with the theoretical best PTA.

## RESULTS

### Demographics

Over the 24-month study period, 63 children with CF had 94 admissions during which they received IV tobramycin for a respiratory exacerbation. This population provided 450 serum tobramycin concentrations; the median number of tobramycin concentrations per child was 4 (range, 1–34). In 29/450 (6%) samples, the concentration was BLQ (<0.6 mg/L). Patient characteristics are described in Table 1. The median age was 13.3 years (range, 0.2–19.3), median weight was 43.8 kg (range, 5.0–81.7), median height was 1.53 m (range, 0.56–1.85), and median creatinine was 43 µmol/L (range, 14–107). The majority of children (54/63, 86%) had CF-related comorbidities, including pancreatic insufficiency (45/63, 71%), diabetes mellitus (15, 24%), and liver disease (15, 24%).

**TABLE 1:** Patient characteristics^*a*^^,^^*b*^

Demographics	N (%) or median (range)
Male n (%)	34/63 (53%)
Age (years)	13.3 (0.2–19.3)
<2 years n (%)	10 (10.6)
2–12 years n (%)	28 (29.8)
>12 years n (%)	56 (59.6)
Weight (kg)	43.8 (5.0–81.7)
Height (m)	1.53 (0.56–1.85)
Serum creatinine (μmol/L)	43 (14–107)
eGFR (mL/min)	127 (58–265)
Comorbidities	
Pancreatic insufficiency	45/63 (71%)
Bronchiectasis	36/63 (57%)
Diabetes mellitus	15/63 (24%)
Liver disease	15/63 (24%)
Allergic bronchopulmonary aspergillosis	8/63 (13%)

^*a*^ eGFR – estimated glomerular filtration rate (calculated using Modified Schwartz Equation).

^*b*^ If the patient experienced >1 admission, the summary of discrete variables (sex, comorbidities) is based on their first admission and the summary of continuous variables (age, weight, height, and serum creatinine) is based on each admission.

### Tobramycin dosing and concomitant medications

Tobramycin dosing details and concomitant medications are outlined in Table 2. The median initial tobramycin dose was 10 mg/kg (range, 0.2–14) OD for a median duration of 12 days (range, 1–26). Tobramycin was administered in combination with another antibiotic in 92/94 (98%) admissions; the most common concomitant antibiotics were piperacillin/tazobactam (45/92, 49%) and ceftazidime (45/92, 49%). Other concomitant medications included CFTR modulators in 34/94 (36%), NSAIDs in 7 (7%), and diuretics (spironolactone and/or furosemide) in 4 (4%), all of which were concomitantly administered during the tobramycin course prior to and throughout TDM. Among the 34 patient admissions in which CFTR modulators were concurrently administered with tobramycin, 16 (47%) received ivacaftor/lumacaftor, 12 (35%) ivacaftor/tezacaftor, 6 (18%) ivacaftor alone, and 3 (8.8%) elexacaftor/tezacaftor/ivacaftor. CFTR modulator therapy had commenced a median of 327 days (range, 1–1,724) prior to the start of the tobramycin course.

**TABLE 2:** Tobramycin dosing information (*n* = 94)^*a*^

Characteristic	N (%) or median (range)
Initial dose (mg/kg)	10 (0.2–14)
Treatment duration (days)	12 (1–26)
Concomitant medication	
β-lactam	92 (98%)
Piperacillin/tazobactam	45 (49%)^*b*^
Ceftazidime	45 (49%)
Meropenem	3 (3.3%)^*b*^
Cefepime	1 (1.1%)
Bactrim	6 (6.4%)
Ciprofloxacin	2 (2.1%)
Vancomycin	1 (1.1%)
CFTR modulator	34 (36%)
Ivacaftor	6 (18%)
Ivacaftor/lumacaftor	16 (47%)
Ivacaftor/tezacaftor	12 (35%)
Elexacaftor/tezacaftor/ivacaftor	3 (8.8%)^*c*^
Non-steroidal anti-inflammatory drugs	7 (7.4%)
Diuretics	5 (5.3%)

^*a*^ CFTR – Cystic fibrosis transmembrane conductance regulator.

^*b*^ Two patient encounters received both tobramycin+meropenem and tobramycin+piperacillin/tazobactam for an equal duration during treatment period.

^*c*^ All also received ivacaftor in the same encounter.

### Tobramycin population pharmacokinetic model

A one-compartment model provided the best description of the data, whereas a two-compartment model led to model instability characterised by high fluctuations in OFV (by 201 points) and was therefore not used. This was parameterized in terms of CL, V, and infusion duration (D_1_). D1 was included as a fitted parameter due to the observational design of the study and the potential inaccuracy in the documented 0.5 h infusion duration. Between-subject variability was considered for all parameters, and the residual variability was modeled as additive and proportional. The final model included size (WT) on CL and V, maturation on CL, and eGFR on CL (Supplementary Equations E1–E5). Changes in OFV following covariate inclusion are detailed in Table S1. The final parameter estimates and relative standard error are displayed in Table 3. Goodness-of-fit plots and visual predictive checks showed a good predictive performance of the model (Fig. S1 and S2). Fat-free mass, serum creatinine, albumin, and concomitant drugs, including CFTR modulators, NSAIDs, and diuretics, were not found to be significant and not included in the final model.

**TABLE 3:** Parameter estimates from the final model^*a*^

Parameter	Final estimate	SE	RSE (%)
CL (L/h)	6.17	0.330	5.35
WT_CL_	0.663	0.049	7.33
eGFR_CL_	0.4 (fixed)	–^*b*^	–
V (L)	15.5	1.08	6.97
WT_V_	0.744	0.068	9.13
D1 (h)	0.801	0.083	10.4
Between-subject variability (standard deviation)			
ω_CL_	0.170	0.029	17.2
ω_V_	0.173	0.025	14.4
ω_D1_	0.404	0.141	35.0
Residual variability			
Additive error	0.173	0.041	23.7
Proportional error	0.103	0.009	9.09

^*a*^ SE – Standard error; RSE – Residual standard error; CL – Clearance; WT_CL_ – exponent for weight on clearance; eGFR_CL_ – exponent for eGFR on clearance; V – Volume of distribution; WT_V_ – exponent for weight on volume; D1 – rate of infusion.

^*b*^ “–” indiactes that there is no SE or RSE measured for this parameter.

### Individualized dosing strategy and probability target attainment of achieving target AUC_24,ss_

Of the 94 admissions, the target AUC_24,ss_ of 80–110 mg/L⋅h was attained in 53 (56%) admissions, with a median AUC_24,ss_ of 91 mg/L⋅h (range, 49–138). Of those children whose AUC_24,ss_ was outside the target range, 28 (30%) were <80 mg/L⋅h, and the remaining 13 (14%) were >110 mg/L⋅h.

The individualized dosing strategy estimates the patient’s CL based on age (years), weight (WT; kg), height (HT; metres), and serum creatinine (CR; mg/dL) using Equation 1. The optimal dose is then calculated using Equation 2 to achieve a target AUC_24,ss_ of 95 mg/L⋅h. It can be accessed here: https://www.kidscalc.org/.


(1) $$CL\left(L/h\right)=6.17\cdot \frac{{\left(\left(AGE\cdot 52\right)+\;40\right)}^{3.4}}{47.7^{3.4}+{\left(\left(AGE\cdot 52\right)+40\right)}^{3.4}}\cdot \left({\left(\frac{WT}{70}\right)}^{0.663}\right)\cdot \left({\left(\frac{41.3*\left(HT/CR\right)}{127.3}\right)}^{0.4}\right)$$

(2) Optimal Dose (mg)=95 ⋅CL

**Fig 1:**
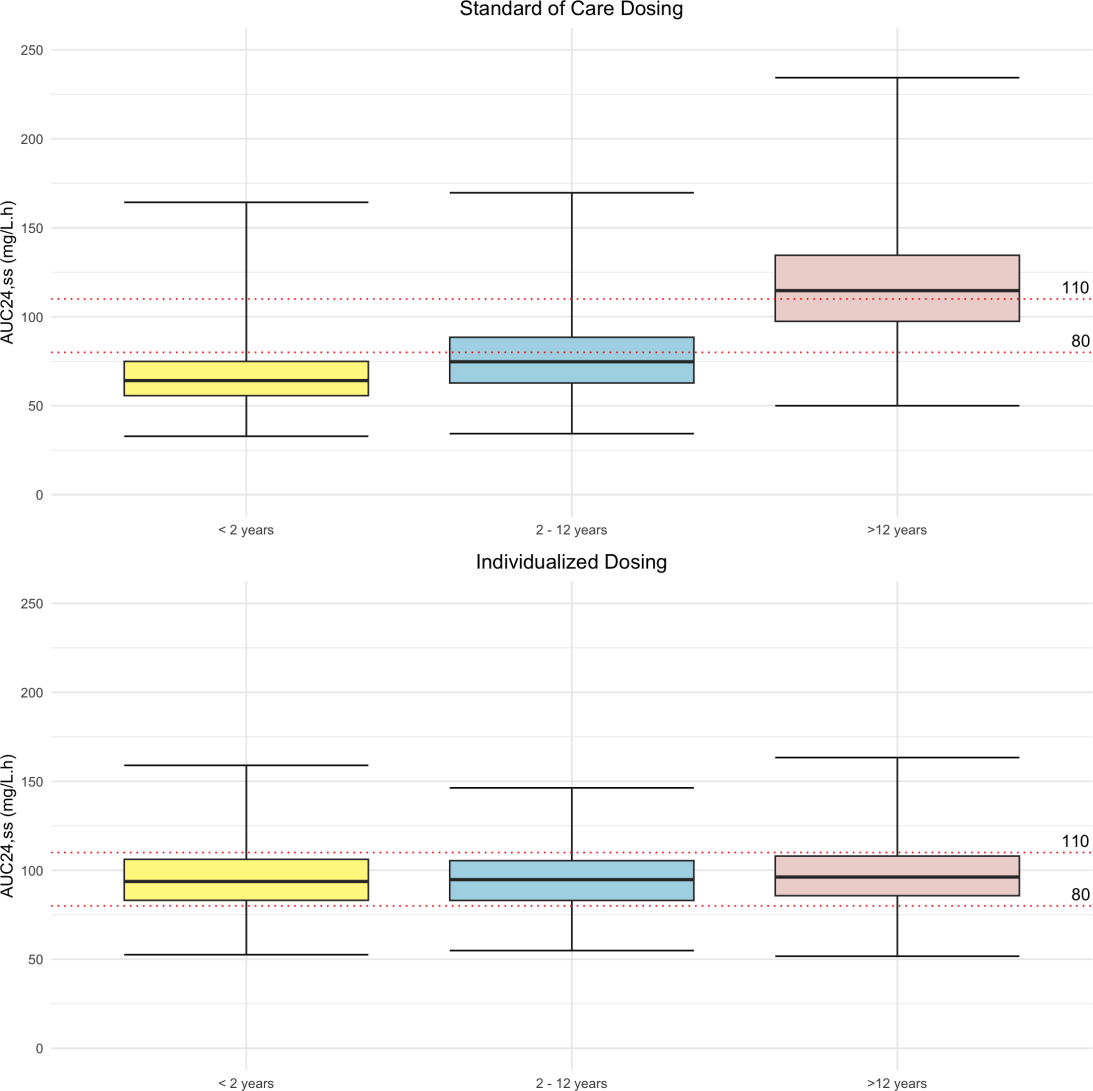
Simulated tobramycin exposure from standard tobramycin dosing (10 mg/kg once daily) versus individualized dosing for different age groups with normal serum creatinine.

From the simulations, the theoretical best PTA for an AUC_24,ss_ of 80–110 mg/L⋅h was 0.64. The PTA in children of all ages using standard dosing compared with the individualized dosing strategy is 0.31 and 0.64, respectively. This, alongside the PTA and the AUC_24,ss_ achieved within each age group, is outlined in Table 4 and Fig. 1, respectively. Overall, with standard dosing of 10 mg/kg OD, only 15% of children aged <2 years achieved the therapeutic target, with 83% having subtherapeutic exposures. Individualized dosing achieved the AUC_24,ss_ target in 62% of children <2 years old, with 19% being underdosed. By contrast, the majority (58%) of children aged >12 years receiving standard dosing exceeded the therapeutic target. This reduced to 23% with individualized dosing. The median dose from the individualized dosing strategy was 11.8 mg/kg (range, 5.3–16) OD, with younger children receiving larger doses by weight, compared with older children (median dose was 15.1, 12.8, and 8.4 mg/kg, for those aged <2, 2–12, and >12 years old, respectively).

**TABLE 4:** PTA of achieving target AUC_24,ss_ 80–110 mg/L⋅h from SOC dosing versus individualized dosing (*n* = 1000)

	Outside target AUC_24,ss_ range (%)		PTA
	<80 mg/L⋅h	>110 mg/L⋅h	
SOC dosing			
All ages	49.4	19.8	0.31
<2 years	83.3	1.9	0.15
2–12 years	60.7	6.6	0.33
>12 years	6.7	57.7	0.36
Individualized dosing			
All ages	16.7	19.3	0.64
<2 years	19.1	19.1	0.62
2–12 years	19.8	17.6	0.63
>12 years	13.7	22.5	0.64

## DISCUSSION

This study shows that only 1/3 of children with CF receiving IV tobramycin are expected to achieve the target AUC_24,ss_ of 80–110 mg/L⋅h with the standard dosing regimen, with younger children aged <2 years having the lowest attainment (15%). By implementing a fully individualized dosing strategy, therapeutic target attainment improved across all groups, with 62% of children <2 years achieving this target range.

The narrow therapeutic target of 80–110 mg/L⋅h is the primary reason for the relatively low PTA. This differs from other antibiotics such as beta-lactams, where the PTAs are reported to be >80%, as these medications are well tolerated, and often, no upper limit for target antibiotic exposure is applied (30–33). Despite the limited number of clinical studies, it is widely accepted that higher tobramycin exposure increases the risk of developing adverse effects, particularly nephrotoxicity and ototoxicity. A study in adult patients receiving IV tobramycin showed that those who developed acute kidney injury had greater cumulative AUC levels during a 72 h period after the first dose compared with those who did not (median AUC_0-72_ was 340.6 mg/L⋅h versus 214.4 mg/L⋅h, respectively; *P* = 0.015) (7). Another study in adult CF patients receiving IV tobramycin found a positive correlation between cumulative tobramycin AUC and hearing loss using a statistical model (34).

In our study, a fully individualized dosing strategy was the most beneficial in children aged <2 years. This is unsurprising as tobramycin is almost exclusively cleared by renal filtration, which is not accounted for in current dosing strategies. These findings are consistent with a retrospective study of 85 children with CF reporting that a dose of 10 mg/kg OD achieved a target AUC_24_ of 80–115 mg/L⋅h in only 44% of encounters (4). The study found that older children were more likely to attain the therapeutic target compared to younger children: the odds of achieving the target AUC_24_ was 56% higher for children aged 10 vs 5 years (*P* = 0.033). Similarly, a retrospective study of children aged <6 years reported that higher doses of 12 mg/kg OD are required to achieve target peak and trough concentrations (35). This may be explained by the negative correlation between V and age reported in a study comparing the PK of tobramycin in adults versus children (36). This change in V with age was primarily attributed to tobramycin distribution in the extracellular fluid, which reduces with increasing age (36). We observed that younger children (born at term) exhibit higher clearance per kg (L/h/kg) compared with adults and older children (Fig. S3). This finding also explains why the PTA following standard dosing is lower in younger children, with the majority experiencing subtherapeutic levels.

Tobramycin pharmacokinetics was best described in this study using a one-compartment PK model, and the final popPK parameter estimates are consistent with other published studies (12, 37). A study of 257 children developed a one-compartment popPK model, which reported a CL of 5.59 L/h and V of 18.9 L, similar to our study (CL, 6.17 L/h and V, 15.5 L) (37). Another study in 35 children also reported similar parameter estimates despite using a two-compartment popPK model (CL, 6.37 L/h, V of the central compartment 18.7 L, V of the peripheral compartment, 1.32 L) (12). Interestingly, in these two studies, WT was the only significant covariate identified and included in their final model. However, the final popPK model in our study included additional covariates (maturation and eGFR), as tobramycin is almost exclusively renally cleared via glomerular filtration. As glomerular filtration is heavily influenced by age, size, and maturation, these covariates were added before the remaining covariates due to the wide range of ages in our study population. Maturation, derived from PMA, was also accounted for in this pediatric population, which captures the physiological maturation of renal function as GFR does not reach adult levels until approximately 2 years of age, thus likely to influence tobramycin CL (27, 38, 39).

Concomitant CFTR modulator use did not affect tobramycin PK in our study. To date, there have been few studies directly exploring the effect of CFTR function on tobramycin PK. Our findings are consistent with a previous pediatric study in 34 children with CF receiving IV tobramycin, in which concomitant CFTR modulator use did not affect tobramycin exposure (40). However, only a small subset of patients was treated with highly effective modulator therapies such as ivacaftor alone or elexacaftor/tezacaftor/ivacaftor. Thus, the potential effect on renal clearance may have been limited. Another study in both adults and children with CF also did not find any evidence to show that loss of CFTR function directly affects renal function or drug PK, as differences could be explained by correcting for other covariates, such as lean body mass (40, 41). Future research should focus on cohorts receiving highly effective modulators to evaluate the influence of modulator therapy on renal clearance and tobramycin exposure.

This study had some limitations; first, all tobramycin dosing and TDM were conducted per routine care. Therefore, the accuracy of documentation of the timing of the dose and sample cannot be guaranteed. However, this was a prospective observational study, and both sites conduct AUC_24_ monitoring with clinical staff educated on the accurate documentation of these data. Second, inter-occasional variability was not accounted for in this study since the majority of patients only had one admission requiring IV tobramycin therapy, with only seven having more than two admissions. As gestational age is not routinely recorded in clinical notes when children have a postnatal age of greater than 1–2 years, a gestational age of 40 weeks was assumed if not otherwise stated. This is unlikely to significantly affect the PK estimates in this study because, at most, it would only result in a 5% difference in GFR (e.g., a 3 year old child born at 26 weeks’ gestation reaches approximately 95% of the GFR of a term-born 3 year old) (42).

Overall, our data highlight the poor attainment of the target AUC_24,ss_ of 80–110 mg/L⋅h in children with CF, with only one-third expected to achieve this target using the current dosing strategy. This was particularly evident in younger children (<2 years). Using a fully individualized dosing approach (https://www.kidscalc.org/), target attainment increased to 64%. However, due to the narrow therapeutic window, approximately one-third of children are still unable to achieve the therapeutic AUC_24,ss_ target, highlighting the need for continued TDM and auditing following implementation. Consistent with previous studies, concomitant CFTR modulator use had a negligible effect on tobramycin pharmacokinetics.

## SUPPLEMENTAL MATERIAL

Supplemental material aac.00278-25-s0001.docx Additional model building details; Table S1; Fig. S1 to S3.

ASM does not own the copyrights to Supplemental Material that may be linked to, or accessed through, an article. The authors have granted ASM a non-exclusive, world-wide license to publish the Supplemental Material files. Please contact the corresponding author directly for reuse.
