# Supplementary material for: Personalized tobramycin dosing in children with cystic fibrosis: an AUC24-guided approach
Source: Antimicrob Agents Chemother. 2025 Jul 23;69(9):e00278-25. doi: 10.1128/aac.00278-25 (PMC12406653; doi:10.1128/aac.00278-25)

**Supplementary material**

The following described how covariates were handled in the model:

1. All continuous covariates (fat-free mass, eGFR, serum creatinine concentration, and serum albumin concentration), except for weight were centred on the median value where, instead, weight was centred on 70kg.
2. All continuous covariate models were constructed as a power function (normalised covariate to the power of an exponent that was estimated). If estimating the exponent did not result in a stable model, the exponent was then fixed to a value near the estimated value to stabilise the model.
3. All categorical covariate (e.g. sex, concomitant medications; NSAIDs, diuretics, and CFTR modulators) models were constructed as a shift from a reference value, e.g. for sex the reference value was 1 for males.
4. Missing covariate values for covariates for which at least one value was available for an individual were imputed by their nearest non-missing value for that individual
5. Missing continuous covariates, where an individual did not have a measured value, were imputed either by the median of the population or based on multivariate regression with other non-missing covariates.
6. Missing categorical covariates were imputed at the mode of the population.

**Final model covariate effects:**

| $CL\left( L/h \right)=\theta_{CL}\cdot MF\cdot\left( \left( \frac{WT}{70} \right)^{\theta_{WT,CL}} \right)\cdot\left( \left( \frac{EGFR}{127.3} \right)^{\theta_{EGFR}} \right)$ | (1) |
| --- | --- |
| $V\left( L \right)=\theta_{V}\cdot\left( \frac{WT}{70} \right)^{\theta_{WT,V}}$ | (2) |
| $D1(h)=\theta_{D1}$ | (3) |
| $MF=\left( \frac{{PMA}^{3.4}}{{47.7}^{3.4}+{PMA}^{3.4}} \right)$ | (4) |
| $EGFR=41.3\cdot\left( \frac{HT}{CR} \right)$ | (5) |

Abbreviations: CL, clearance; WT, weight (kg); EGFR, estimated glomerular filtration rate (Modified Schwartz equation); V, volume of distribution; D1, infusion time (h); MF, maturation function; PMA, post menstrual age; HT, height (m); CR, serum creatinine clearance (mg/dl).

The final parameter estimates are provided in Table 3 of the paper

**Supplementary Table 1:**

| **Covariate** | **Model** | **Reduction in OFV compared to base 1cpt model with combination error** |
| --- | --- | --- |
| D1 parameter | - | 45.5 |
| D1 parameter  Maturation function | -  Maturation on clearance | 60.0 |
| D1 parameter  Maturation function  Size (WT) | -  Maturation on clearance  Size on clearance (fixed exponent) | 112.4 |
| D1 parameter  Maturation function  Size (WT) | -  Maturation on clearance  Size on clearance and volume of distribution (estimated exponent) | 187.2 |
| D1 parameter  Maturation function  Size (WT)  eGFR | -  Maturation on clearance  Size on clearance and volume of distribution (estimated exponent)  eGFR on clearance (fixed exponent) | 201.9 |

**Supplementary Figure 1:** Goodness of fit plots


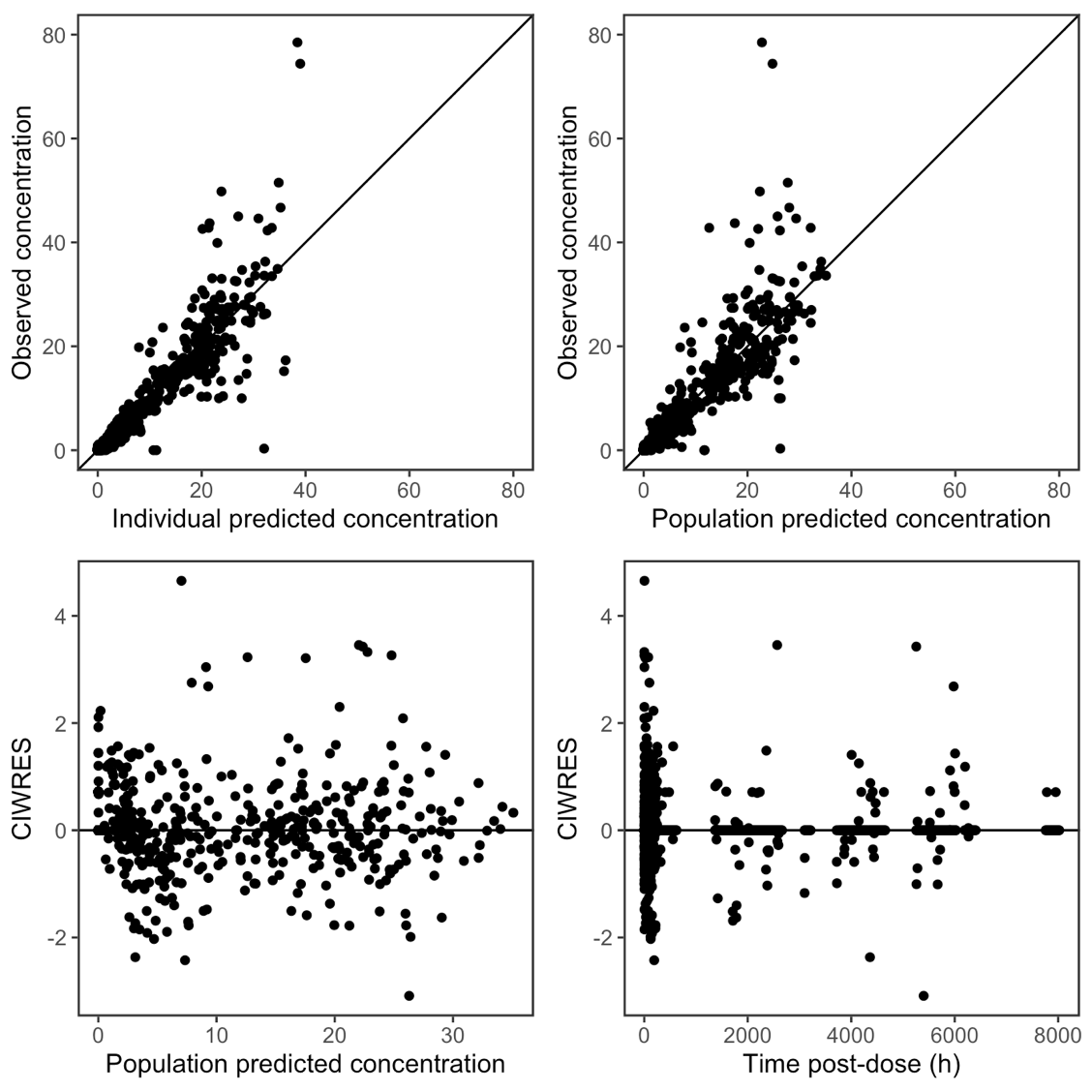


**Supplementary Figure 2:** Standard visual predictive check (A) and prediction-corrected visual predictive check (B) of the tobramycin population pharmacokinetic model. The open circles are observed concentrations. The dashed lines are the 10th and 90th percentiles of observed data and the solid line is the 50th percentile. The bands are the 95% CI of the percentiles of the model predictions from the tobramycin pharmacokinetic model.

**Supplementary Figure 3:** Average clearance per kg by age in children. Clearance was determined using a simplified version of Equation 1 from the paper, excluding the eGFR covariate, and was divided by weight to calculate clearance per kilogram. Clearance per kilogram was analysed for children aged 12 weeks to 2 years (A) and for all children (B). Median weight for each age group was derived from the WHO growth chart. The dotted horizontal line represents the clearance per kilogram for a 70kg adult. This shows that children exhibit higher clearance per kg (L/h/kg) compared to adults and older children.

**A**


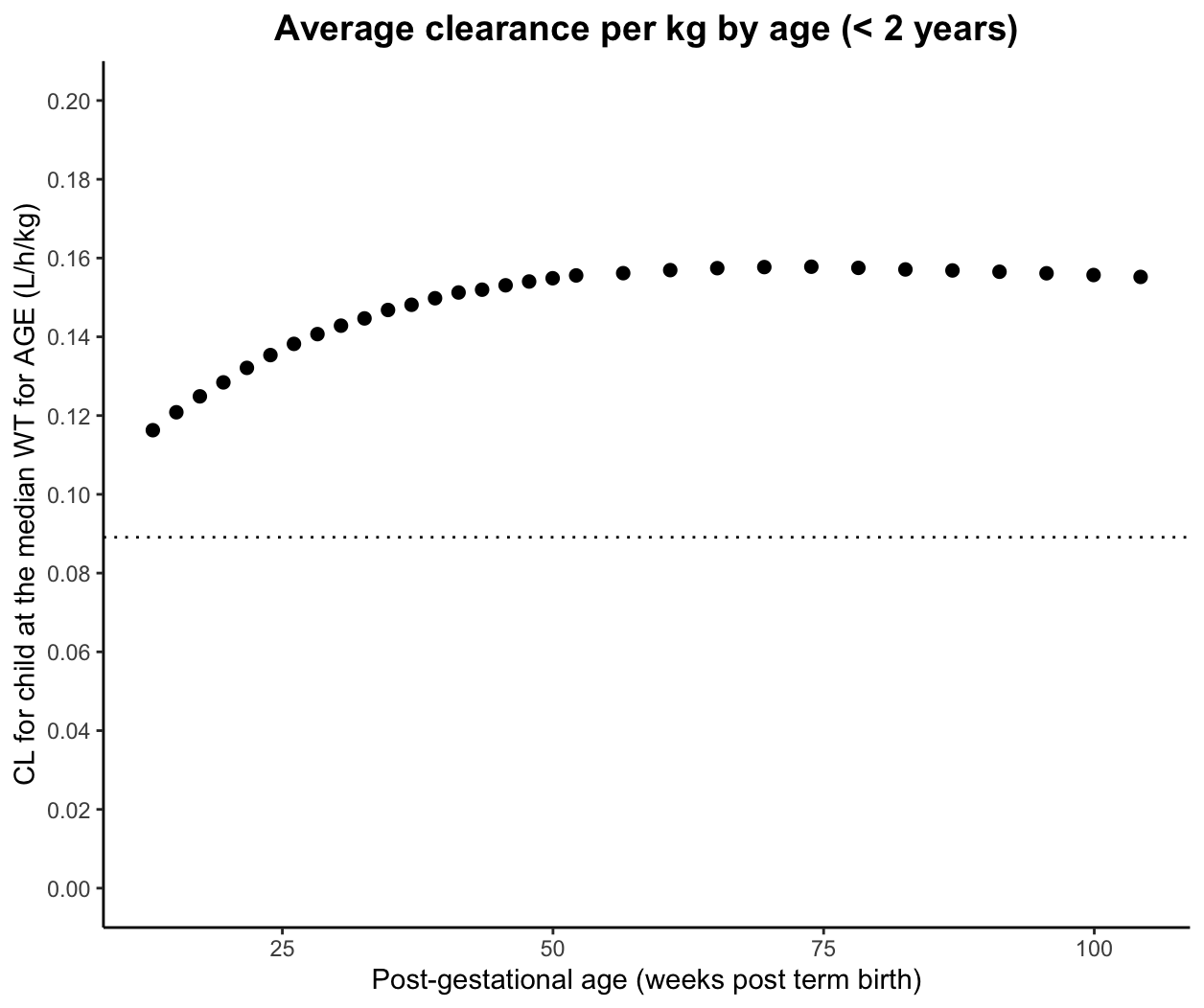


**B**


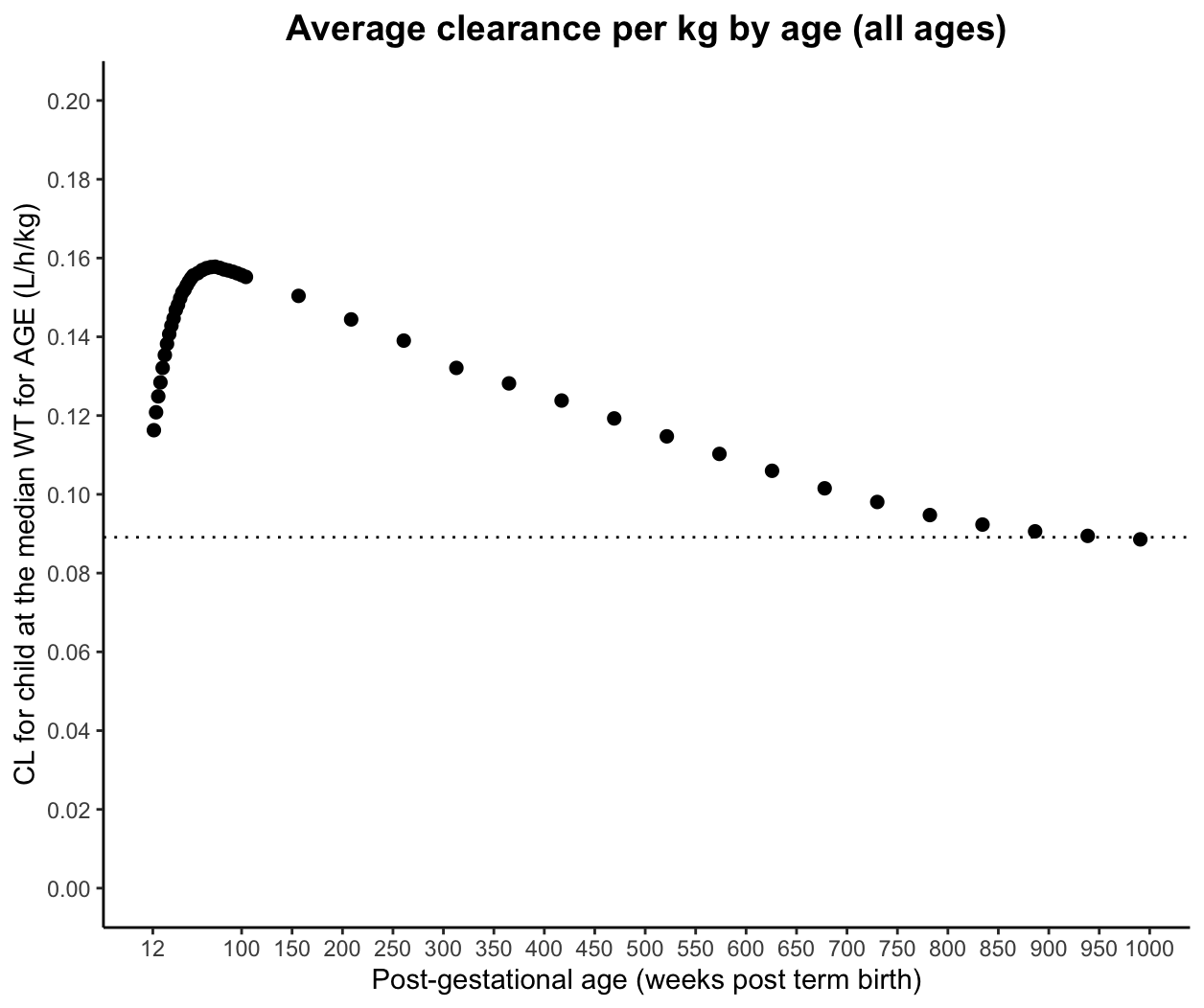

Supplement: Supplemental material — Additional model building details; Table S1; Fig. S1 to S3. [file aac.00278-25-s0001.docx]
